# Performance and Nanostructure Simulation of Phosphogypsum Modified by Sodium Carbonate and Alum

**DOI:** 10.3390/ma14195830

**Published:** 2021-10-05

**Authors:** Dongqing Zhong, Jingchen Wang, Guihua Hou, Luming Wang, Qian Wu, Bao Lu

**Affiliations:** 1 School of Materials Science and Engineering, Yancheng Institute of Technology, Yancheng 224051, China; zdq811004@ycit.edu.cn (D.Z.); houguihua@ycit.cn (G.H.); wlm@ycit.cn (L.W.); wysqh1001@163.com (Q.W.); 2 Phillips Exeter Academy, Exeter, NH 03833, USA; wangjingchen028@163.com

**Keywords:** phosphogypsum, sodium carbonate, alum, compound doping

## Abstract

This paper presents a new modification of the nanostructure of CaSO_4_·2H_2_O crystals containing nanopores. This nanoporous structure was achieved in phosphogypsum samples that were modified by sodium carbonate and alum. The effects of sodium carbonate and alum on the properties of phosphogypsum were studied. X-ray diffraction (XRD) and scanning electron microscopy (SEM) methods were used to explore the micro-mechanism of the composite system. Subsequently, molecular dynamics simulations were used to study the nanopore structures of the modified CaSO_4_·2H_2_O. The results show that the addition of sodium carbonate and alum reduced the absolute dry density by 23.1% compared with the original phosphogypsum sample, with a bending strength of 2.1 MPa and compressive strength of 7.5 MPa. In addition, new hydration products, sodium sulfate and sodium aluminum sulfate, were formed in the sample doped with sodium carbonate and alum. A new nanostructure of CaSO_4_·2H_2_O crystal containing nanopores was formed. Molecular simulations show that the hydration products were responsible for the surface nanopore formation, which was the main factor leading to an increase in mechanical strength. The presented nanopore structure yields lightweight and high strength properties in the modified phosphogypsum.

## 1. Introduction

Phosphogypsum is produced in the chemical process of preparing phosphoric acid using the sulfuric acid method. For every ton of phosphoric acid produced, about 4.5–5.5 tons of phosphogypsum is produced [1,2,3]. Reports show that the average annual emission of phosphogypsum has reached 70 million tons and that the accumulated stockpiles have reached more than 300 million tons while the utilization rate is only about 15% [4,5]. Phosphogypsum storage not only occupies much land but also contains phosphorus [6,7,8,9,10], fluorine [11,12], organic matter [13,14], and other impurities. Once leaching, it pollutes the surrounding soil and water sources and restricts sustainable development. Thus, research on the reuse of phosphogypsum is beneficial to environmental protection.

Phosphogypsum is commonly made into construction and building materials [15,16,17,18], which allows it to become utilized as a resource. Blocks, plasterboard, binders, and self-leveling plaster are the potential phosphogypsum products. Recent researches show significant interest in reducing the absolute dry density of gypsum and other cement-based materials and in simultaneously yielding lightweight and high strength properties [19,20,21]. Examples of treating methods include CO_2_ curing [22], replacement of anhydrite with limestone powder [23], and pretreating lightweight aggregates with soaking and vacuum saturation [24]. For phosphogypsum, this provides a wide range of low-cost applications, which can replace cement products and reduce CO_2_ emissions.

The common method to realize the purpose of lightweight and high strength is by adding air-entraining agent during the mixing process of phosphogypsum. This reduces the absolute dry density of phosphogypsum blocks by increasing the air content of phosphogypsum. Commonly used air-entraining agents are mostly animal and plant air-entraining agents [25,26], ammonium bicarbonate [27,28], hydrogen peroxide [29,30], sodium bicarbonate [31,32], etc. Xiancai Liu [33] added the chemical air-entraining agent polyisocyanate to the construction gypsum, resulting in a significant swelling of the gypsum, and an increase of volume; Alena et al. [34] used the mechanism of the chemical reaction between calcium carbonate and aluminum sulfate to produce carbon dioxide and prepared gypsum with an absolute dry density of (586 ± 19) kg/m^3^, which effectively reduced its absolute dry density. The air-entraining agent reacts with gypsum and then changes its crystal structure. The microstructure of such samples is described as classic lapping of the perfect gypsum crystal with mesoscale pores. The mechanism of air-entraining agents yields more formation of pores in hardened gypsum plaster. In theory, the nanostructure of CaSO_4_·2H_2_O crystal with nanopores can further decrease the dry density of gypsum. 

The studies mentioned in the above paragraph [33,34] have revealed the effect of air-entraining agents. However, they fail to introduce nanopores into the CaSO_4_·2H_2_O crystals. Such modifications regarding nanopores have been made in cement-based materials such as in the stacking process of calcium silicate hydrate [35]. However, studies on the performance and mechanism of phosphogypsum modified by this new nanostructure of CaSO_4_·2H_2_O crystal that contains nanopores are still lacking.

This paper introduces a new modification of the nanostructure of CaSO_4_·2H_2_O crystals containing nanopores. Sodium carbonate and alum were added to phosphogypsum samples for modification purposes. The effect of sodium carbonate and alum content and water–cement ratio on the setting times, mechanical properties, and dry densities of the phosphogypsum samples were studied. The formation of nanopores was observed and the mechanisms of their nanostructures were subsequently studied by molecular dynamics simulations. Molecular simulations were also used to study the enhancing effect of nanopores on the mechanical properties of CaSO_4_·2H_2_O crystals. 

## 2. Materials and Methods

### 2.1. Raw Materials

Phosphogypsum: purchased from Nantong Oasis Energy Conservation and Environmental Protection Products Co., Ltd. (Nantong, China); sodium carbonate: industrial grade, purity 99%, purchased from Shanxi Xinghua Group Co., Ltd. (Xianyang, China); Alum: chemically pure, purity 99.9%, purchased from Lianyungang Guansu Industrial Co., Ltd (Lianyungang, China).

The main component of phosphogypsum is CaSO_4_·0.5H_2_O, and it also contains a small amount of CaSO_4_·2H_2_O and impurities. According to the GB/T 17669.3-1999 test of the mechanical properties of phosphogypsum, when the water–cement ratio is 0.7, its flexural strength is 2.4 MPa and the compressive strength is 8.6 MPa, the absolute dry density of phosphogypsum is 1289 kg/m^3^.

### 2.2. Sample Preparation

**Figure 1:**
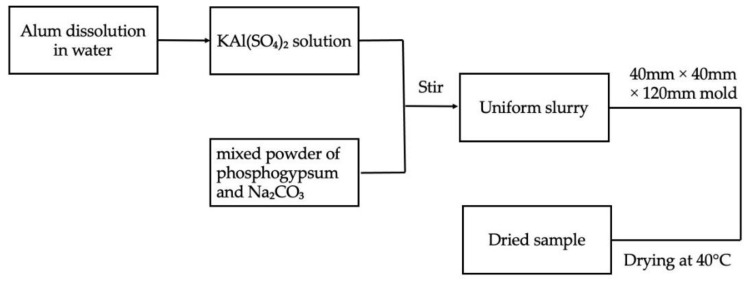
Sample preparation method demonstration.

According to the ratio shown in Table 1, alum was first dissolved in water to make a potassium aluminum sulfate solution; then, phosphogypsum and sodium carbonate were mixed uniformly to make a mixed powder. The mixed powder was poured into the potassium aluminum sulfate solution and quickly stirred until the slurry was uniform, and the slurry was poured into a mold of 40 mm × 40 mm × 120 mm. Then, it was placed in a drying oven at 40 °C until it was completely dry. The sample preparation process is demonstrated in Figure 1. 

### 2.3. Test Methods

The mechanical properties of the sample were tested based on GB/T 17669.3-1999. The initial and final setting time was tested by the Vicat meter per GB/T 17669.1-1999. The domestic Y-550 X-ray diffractometer (XRD, Fangyuan, Liaoning, China) was used to determine the hydration products of the samples. The microscopic morphology of the sample was observed with the QUANT 200 scanning electron microscope of the FEI company in Hillsboro, Oregon, USA. 

### 2.4. Molecular Dynamics Simulation Methods

The large-scale Atomic/Molecular Massively Parallel Simulator (LAMMPS) was used for the molecular dynamics simulations and calculations of CaSO_4_·2H_2_O, where the pore formation mechanism with hydration products as well as the effects of pores on the mechanical properties of the gypsum crystal was studied [36]. CaSO_4_·2H_2_O crystal belongs to the monoclinic crystal system, and its lattice parameters are a = 5.678 Å, b = 15.213 Å, c = 6.286 Å, α = γ = 90°, and β = 114.08° [37]. Based on these parameters, the initial model of CaSO_4_·2H_2_O crystals was established. The COMPASS force field (Condensed-phase Optimized Molecular Potential for Atomistic Simulation Studies) was used in molecular simulations of CaSO_4_·2H_2_O [38]. The force field model includes cross-coupling reactions that predict the vibration frequency and structural changes as well as non-bonding interactions based on the Lennard-Jones-9-6 function and the Coulomb function calculations. The COMPASS force field is widely used in molecular simulation research of gypsum. Chang et al. used the COMPASS force field to study the wettability of the gypsum surface nanosphere [39]. Khalkhali et al. used the COMPASS force field to study the bulk and surface properties of gypsum [40].

#### 2.4.1. Simulation of Pore Formation Mechanisms

To increase the number of crystal grains that can be studied, a 5 Å × 5 Å × 5 Å supercell was first established. Subsequently, a vacuum layer with a width of 5 Å was established to prevent periodic atoms from affecting the reaction surface [41]. Both sides of the vacuum layer channel are wrapped by gypsum crystals to allow for filling of the hydration products. Subsequently, the Metropolis calculation method from the Monte Carlo method was used to adsorb water molecules and hydration products in the gypsum channel [42]. The Monte Carlo method is widely used for water absorption of microporous and mesoporous materials [43,44,45]. In this process, the adsorbed molecules are randomly added, deleted, moved, and rotated and are added to the model through the repeated sampling method of Metropolis, and the energy is minimized. All models used an isothermal and pressure NPT ensemble for dynamics simulation and stability, with a time step of 1 fs and a total time of 1000 ps. The temperature was set to 298 K, and the Nose–Hoover thermostat and Parrinello–Rahman barostat were used to monitor and adjust the temperature, pressure, energy, etc. to maintain the stability of the system. At the same time, the Ewald method is used to calculate static electricity, and the atom-based method was used to calculate van der Waals forces. When the system stabilized, the formation mechanism of pores inside the channel surfaces was studied. 

#### 2.4.2. Effects of Pores on Mechanical Properties 

Taking the center of the outer surface of the gypsum model crystal lattice as the center, hemispherical holes with the diameters of 2 Å, 3 Å, 4 Å, 5 Å, and 6 Å were dug out. Subsequently, water molecules and hydration products were adsorbed on the surface of the pores, and the uniaxial young’s modulus was calculated. In order to complete the uniaxial tensile test, the simulated box was stretched in a single direction under constant strain conditions, and the NPT ensemble was used to ensure that the pressure in other directions remained zero [46,47,48].

## 3. Results and Discussion

### 3.1. Setting Time

**Figure 2:**
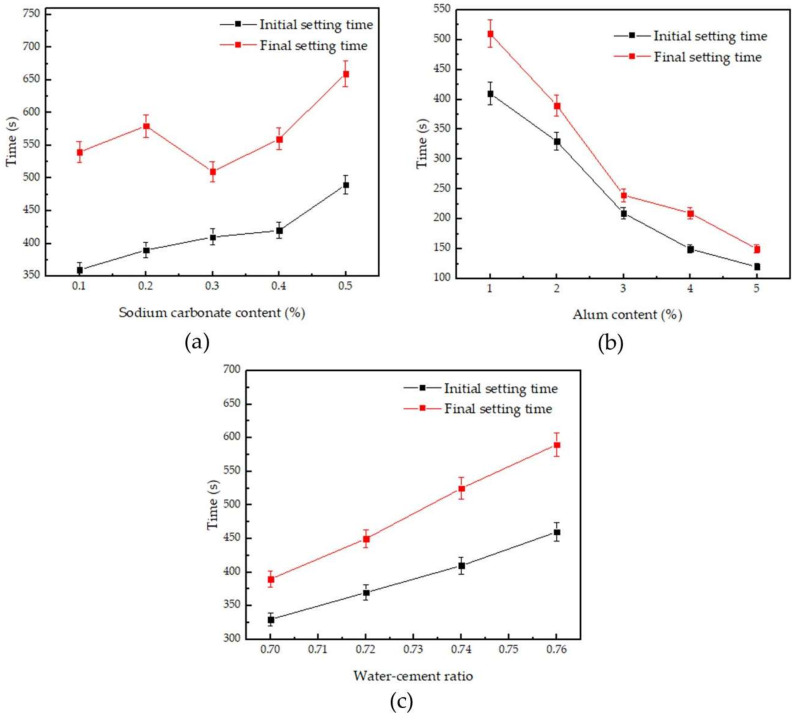
Effect of sodium carbonate and alum on the initial setting time and final setting time: (**a**) alum content is 1%, and sodium carbonate content changed; (**b**) sodium carbonate content is 0.3%, and alum content changed; and (**c**) sodium carbonate content is 0.3%, alum content is 2%, and the water–cement ratio changed.

Table 2 shows the effect of different content of sodium carbonate and alum on the properties of phosphogypsum. Figure 2 displays the effect of sodium carbonate and alum compounding on the initial and final setting times. It can be seen from Table 2 and Figure 2 that, with the increase in sodium carbonate content, the initial setting time and final setting time of phosphogypsum increased; with the increase of alum content, the initial setting time and final setting time of phosphogypsum decreased; and with the increase of water–cement ratio, the initial setting time and final setting time of phosphogypsum linearly increase. The hydration process of gypsum consists of the dissolution of CaSO_4_·0.5H_2_O and the crystallization of CaSO_4_·2H_2_O, and the addition of salt affects the process [49]. Guangya Zheng discussed the retardation mechanism of gypsum and believed that the coating material formed on the crystal surface can delay the hydration of gypsum crystals [50]. When the sodium carbonate content increases, the carbonate ions in the solution and the calcium ions dissolved in the phosphogypsum combine to form calcium carbonate, and the resulting calcium carbonate surrounds the phosphogypsum, which increases the initial setting time and the final setting time of the phosphogypsum. When the content of alum increases, the alum solution contains more sulfate ions, which combine with calcium ions in the solution to form CaSO_4_·2H_2_O, which leads to a decrease in the initial setting time and final setting time of phosphogypsum. When sodium carbonate is mixed with alum, there is a competition mechanism for the influence of the two substances on the setting time of gypsum. The final result is related to the relative ratio of sodium carbonate and alum. Another explanation for the phenomena is relative with impurities in phosphogypsum. The salts added in system may be affected by organic phase or phosphate.

### 3.2. Mechanical Properties

**Figure 3:**
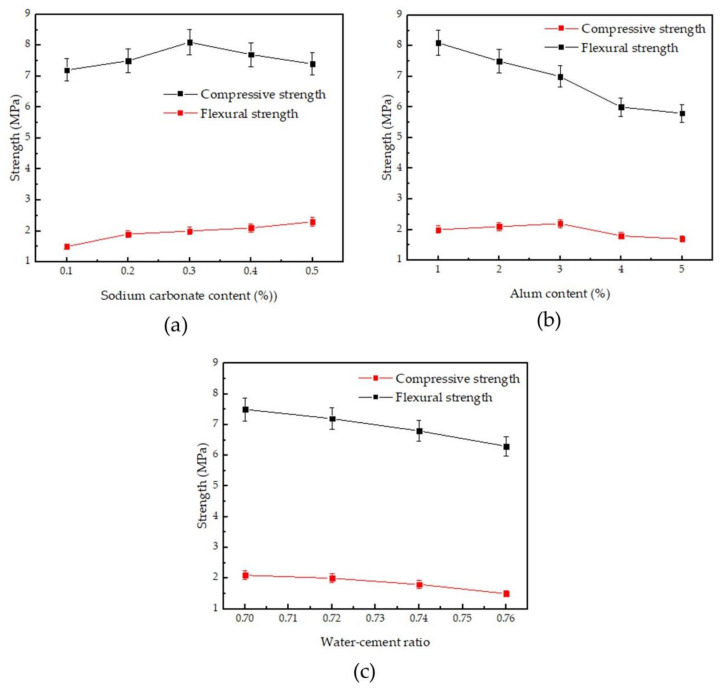
Effect of sodium carbonate and alum on compressive strength and flexural strength: (**a**) alum content is 1%, and sodium carbonate content changed; (**b**) sodium carbonate content is 0.3%, and alum content changed; and (**c**) sodium carbonate content is 0.3%, alum content is 2%, and water–cement ratio changed.

Figure 3 shows the changes in compressive strength and flexural strength of phosphogypsum. It can be seen from Table 2 and Figure 3 that the alum content was first fixed at 1%, and the influence of sodium carbonate content on strength was studied. With the increase of sodium carbonate content, the flexural strength of phosphogypsum gradually increased, and the compressive strength first increased and then decreased. When the sodium carbonate content was 0.3%, the compressive strength reached the peak value of 8.1 MPa. Then, the sodium carbonate content was fixed at 0.3%, and the influence of alum content on the strength was studied. As the alum content increased, the flexural strength of phosphogypsum first increased and then decreased, and the compressive strength gradually decreased. Specifically, the compressive strength of phosphogypsum was gradually reduced from 8.1 MPa to 6.8 MPa, and when the alum content was 3%, the flexural strength reached the peak value of 2.2 MPa. The effect of the water–cement ratio on strength was subsequently studied. The sodium carbonate content was fixed at 0.3%, and the alum content was fixed at 2%. With the increase in the water–cement ratio, the flexural strength and compressive strength of phosphogypsum show a linearly decreasing trend. In order to obtain phosphogypsum with high mechanical properties, the water–cement ratio should be controlled within the range of 0.70–0.72. At this time, the flexural strength is higher than 2.0 MPa, and the compressive strength is higher than 7.2 MPa. When sodium carbonate and alum are compounded, the flexural strength and compressive strength of the modified phosphogypsum are slightly lower than that of the blank phosphogypsum under the same water–cement ratio. To summarize the effects of sodium carbonate, alum, and water–cement ratio on the mechanical properties of phosphogypsum, when the sodium carbonate content was 0.3%, the alum content was 1%, and the water–cement ratio was 0.70, the mechanical properties of the modified phosphogypsum were optimal. The flexural strength was 2.0 MPa, and the compressive strength was 8.1 MPa. Theoretically, the strength is relative to the pore structure and CaSO_4_·2H_2_O crystals. For example, the strength of α type CaSO_4_·2H_2_O crystals is dozens of times higher than β type CaSO_4_·2H_2_O crystals. The crystal morphology causes the change of gypsum strength. Thus, modification about CaSO_4_·2H_2_O crystals provide a “bottom-up” method to enhance the gypsum strength. In our research, the nanopores were introduced into the β type CaSO_4_·2H_2_O crystals. This provides a better strength of β type CaSO_4_·2H_2_O crystals, which is discussed below.

### 3.3. Absolute Dry Density

**Figure 4:**
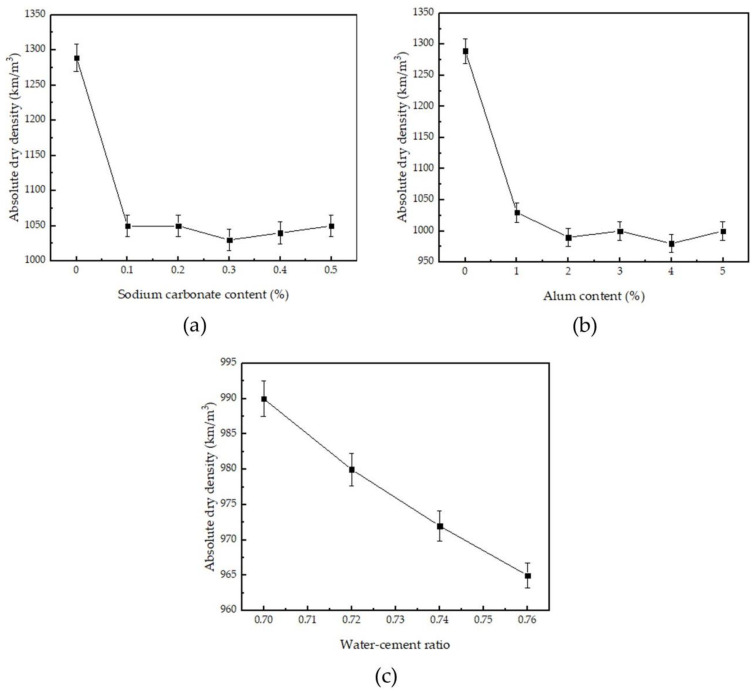
Effect of sodium carbonate and alum on the absolute dry density of phosphogypsum: (**a**) alum content is 1%, and sodium carbonate content changed; (**b**) sodium carbonate content is 0.3%, and alum content changed; and (**c**) sodium carbonate content is 0.3%, alum content is 2%, and water–cement ratio changed.

Figure 4 shows the influence of sodium carbonate and alum on the absolute dry density of phosphogypsum. It can be seen from Figure 4 and Table 2 that the blank phosphogypsum sample G_0_ with a water–cement ratio of 0.70 has a bone-dry density of 1289 kg/m^3^, and the blank phosphogypsum sample G_1_ with a water–cement ratio of 0.88 has a bone-dry density of 998 kg/m^3^. When the water–cement ratio is 0.70, the absolute dry density of phosphogypsum mixed with sodium carbonate and alum is maintained at 980–1050 kg/m^3^; when the water–cement ratio is increased to 0.74, the absolute dry density of phosphogypsum linearly decreases. The absolute dry density of phosphogypsum is in the range of 960–990 kg/m^3^, indicating that the compounding of sodium carbonate and alum causes the absolute dry density of phosphogypsum to decrease. Comparing the mechanical properties of blank phosphogypsum G_1_ with sample P_1_ of similar dry density with sodium carbonate and alum added, it can be found that the flexural strength of P_1_ is about 123.5% of that of G_1_, and the compressive strength of P_1_ is about 110.3% of that of G_1_. Compounding sodium carbonate and alum can improve the mechanical properties of phosphogypsum and can reduce the lowering of mechanical properties due to air entraining.

### 3.4. XRD Analysis 

**Figure 5:**
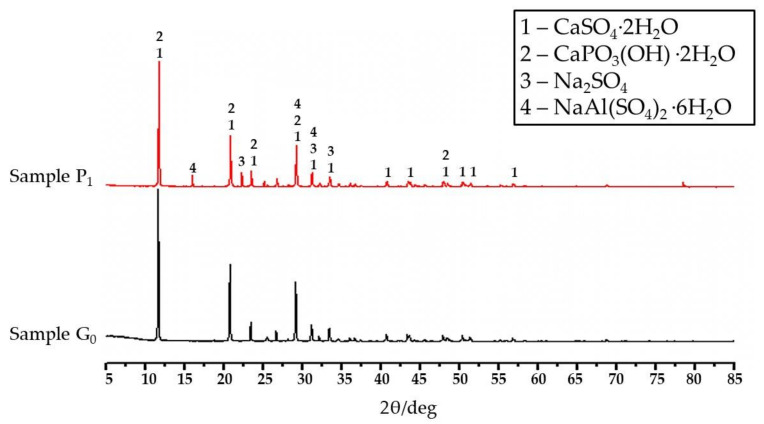
XRD diffraction image of phosphogypsum.

Figure 5 shows the XRD diffraction images of modified phosphogypsum and blank phosphogypsum. It can be seen from Figure 5 that the hydration products of the blank group phosphogypsum G_1_ are CaSO_4_·2H_2_O and CaPO_3_(OH)·2H_2_O. The hydration products of phosphogypsum P_1_ mixed with calcium carbonate and alum are CaSO_4_·2H_2_O, CaPO_3_(OH)·2H_2_O, Na_2_SO_4_, and the NaAl(SO_4_)_2_·6H_2_O. XRD results showed that new hydration products were formed after modification: NaAl(SO_4_)_2_·6H_2_O and Na_2_SO_4_. The test of bone-dry density and mechanical properties showed that the phosphogypsum added with calcium carbonate and alum reduced the bone-dry density and enhanced the mechanical properties. This phenomenon may be related to the effects of the newly formed hydration products NaAl(SO_4_)_2_·6H_2_O and Na_2_SO_4_.

### 3.5. SEM Morphology 

**Figure 6:**
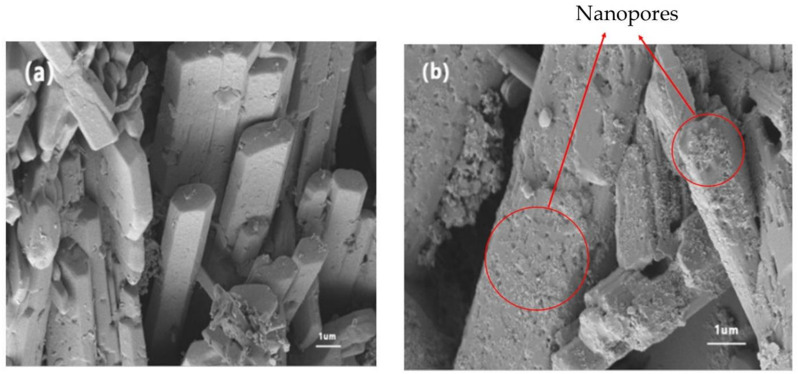
SEM image of phosphogypsum. (**a**) G_0_: blank group; (**b**) P_1_: modified group.

Figure 6 shows the SEM image of phosphogypsum. Figure 6a is the SEM image of the blank group phosphogypsum; Figure 6b is the SEM image of the phosphogypsum mixed with calcium carbonate and alum. It can be seen from Figure 6a that the phosphogypsum CaSO_4_·2H_2_O crystals of the blank group are closely arranged in a prismatic shape, and the surface is smooth and has no obvious defects. It can be seen from Figure 6b that the CaSO_4_·2H_2_O crystals of phosphogypsum P_1_ mixed with calcium carbonate and alum are prismatic. In addition, there are nanopores in the CaSO_4_·2H_2_O crystals. The formation of nanopores may be one of the factors leading to the enhancement of mechanical properties. In order to explore the influence of pores on the mechanical properties of gypsum, molecular dynamics simulation was used to study the influence of nanometer size and pores on the mechanical properties of CaSO_4_·2H_2_O crystals.

### 3.6. Molecular Dynamics Simulation Analysis

**Figure 7:**
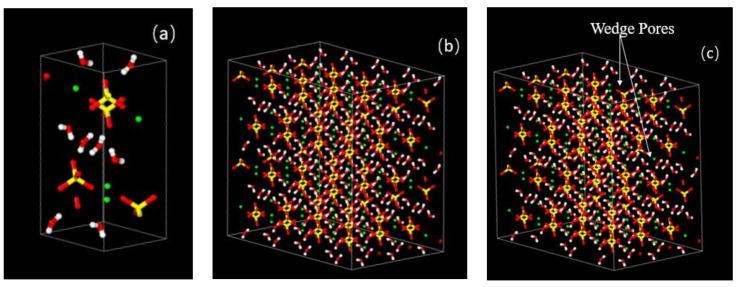
CaSO_4_·2H_2_O modeling process (**a**) unit crystal; (**b**) 4 Å × 2 Å × 3 Å supercell; (**c**) modified crystal with wedge pores. (White balls are hydrogen, red balls are oxygen, yellow balls are sulfur, and green balls are calcium.)

**Figure 8:**
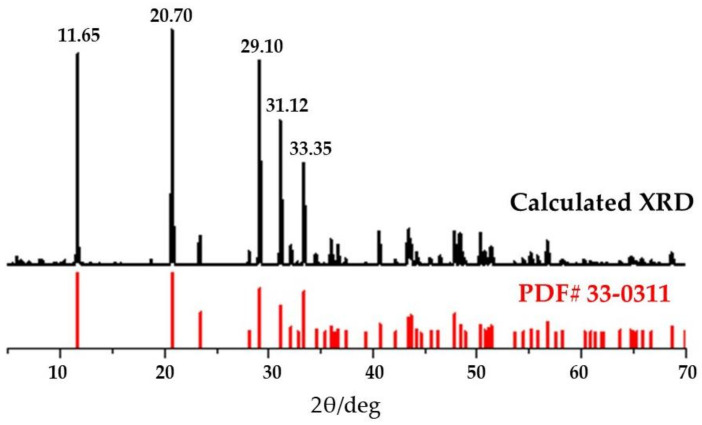
XRD pattern of the CaSO_4_·2H_2_O model.

On the perfect crystal form of CaSO_4_·2H_2_O, wedge-shaped pores were made to conform to the real situation of CaSO_4_·2H_2_O crystals. Figure 7 shows the modeling process of CaSO_4_·2H_2_O, where wedge pores were created to conform to the realistic mechanical properties. The uniaxial young’s modulus of the constructed model is 0.73 GPa, which is consistent with the experimental test [51]. Figure 8 is the calculated XRD pattern of the CaSO_4_·2H_2_O model. It can be seen from Figure 8 that the main characteristic peaks of the calculated XRD pattern of the constructed model are consistent with the peak positions of the XRD pattern of the experimentally tested CaSO_4_·2H_2_O (PDF# 33-0311), indicating that the CaSO_4_·2H_2_O model established in this paper is consistent with that of experimental preparation.

#### 3.6.1. Pore Formation Mechanisms

**Figure 9:**
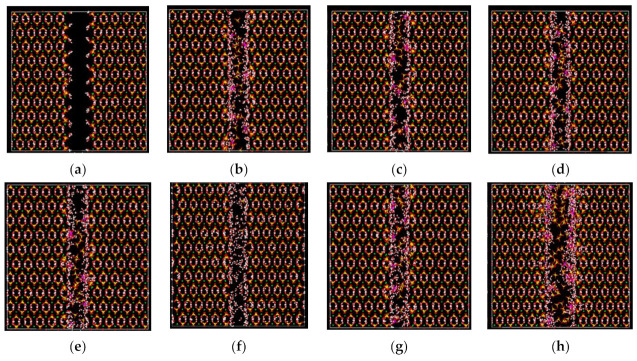
CaSO_4_·2H_2_O model with hydration product adsorption: (**a**) model with G_0_ contents, (**b**) model with F_1_ contents, (**c**) model with F_2_ contents, (**d**) model with F_3_ contents, (**e**) model with F_4_ contents, (**f**) model with P_1_ contents, (**g**) model with P_2_ contents, and (**h**) model with P_3_ contents. (White balls are hydrogen, red balls are oxygen, yellow balls are sulfur, green balls are calcium, purple balls are sodium, and pink balls are aluminum).

**Figure 10:**
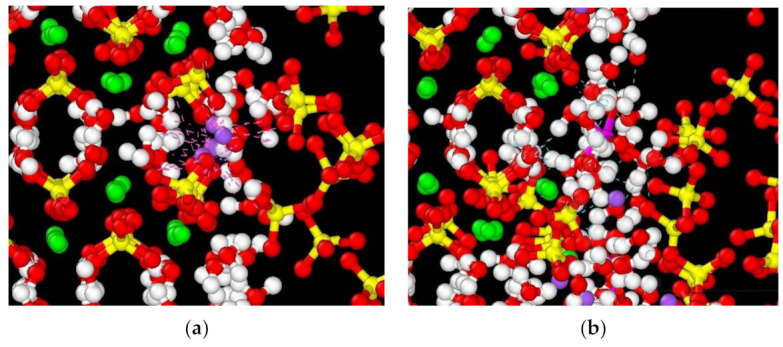
Interactions between hydration products and CaSO_4_·2H_2_O surface: (**a**) Na ion interactions; (**b**) sodium aluminum sulfate interactions. (White balls are hydrogen, red balls are oxygen, yellow balls are sulfur, green balls are calcium, purple balls are sodium, and pink balls are aluminum).

In order to further explore the effects of nanopores on CaSO_4_·2H_2_O, molecular dynamics simulation was used to study the mechanism regarding the formation of the nanopores on the surface of columnar CaSO_4_·2H_2_O crystals produced by the hydration products Na_2_SO_4_ and NaAl(SO_4_)_2_·6H_2_O. Figure 9 shows the CaSO_4_·2H_2_O models with different contents of salts. It can be seen from the figure that the CaSO_4_·2H_2_O model without added hydration products is stable, and surface water molecules escape outward. The surface of the CaSO_4_·2H_2_O channel after the addition is deformed, and the hydration products can be adsorbed on the surface of the CaSO_4_·2H_2_O. The sulfate groups on the surface undergo more obvious rotational and translational displacement, and the surrounding calcium atoms also undergo similar changes. Table 3 displays the total surface pore volume (including channel volume) of the original model and the modified models. It can be seen from the table that the pore volume of CaSO_4_·2H_2_O without addition is the smallest, but after adding hydration products, the pore volume on the CaSO_4_·2H_2_O surface increases. This indicates that microscopic pores formed on different sites of the channel surface. Among them, the pore diameter ranges from approximately 2 Å to 6 Å. When the contents of Na_2_SO_4_ and NaAl(SO_4_)_2_·6H_2_O are 0.3% and 3%, respectively, the pore volume is the largest. Figure 10 shows the interaction between hydration products and CaSO_4_·2H_2_O. Figure 10a shows the interaction between Na ions and the surface of the pores. It can be seen from Figure 10a that the interaction forces are dominated by the Van der Waals forces between Na ions and sulfate groups on the CaSO_4_·2H_2_O surface, resulting in microscopic deformation of around two–three sulfate groups on the surface. Figure 10b shows the interaction between NaAl(SO_4_)_2_·6H_2_O and the surface of CaSO_4_·2H_2_O. It can be seen from Figure 10b that the hydrated product exists in a crystalline structure, and its surface water molecules form about 20 hydrogen bonds with the sulfate groups and water molecules of CaSO_4_·2H_2_O and, at the same time, cause microscopic deformation of around five–six sulfate groups. It can be seen from the figure that Na ions can penetrate deeper into the CaSO_4_·2H_2_O surface and cause structural changes. In addition, due to the strong interaction forces between sodium aluminum sulfate and water molecules, more surrounding water molecules are attracted, of which about five water molecules are provided by CaSO_4_·2H_2_O after erosion. Mao et al. used molecular dynamics simulations to study the interactions of additives on the CaSO_4_·0.5H_2_O surface [52]. They discovered that the interaction energy is determined by the electrostatic attraction between the O in the additive (-0.706 e) and the Ca^2+^ ions as well as the repulsion between the S in the additive (+1.318 e) and Ca^2+^. In this experiment, the Na^+^ ions experience a similar magnitude of electrostatic attraction to the O in the sulfate of CaSO_4_·2H_2_O (−0.879 e), while experiencing a much smaller repulsion with the S in the sulfate of CaSO_4_·2H_2_O (+1.516 e). This shows that hydration products, especially Na^+^ ions, can form strong interaction forces with the surface of CaSO_4_·2H_2_O. This causes the surface structure deformation of CaSO_4_·2H_2_O, which is the main mechanism in the formation of pores. The formation of surface nanopores is correlated with the observed increase in mechanical properties. The extent of such effects is presented in the next section. 

#### 3.6.2. Effects of Pore Diameter on Mechanical Properties

**Figure 11:**
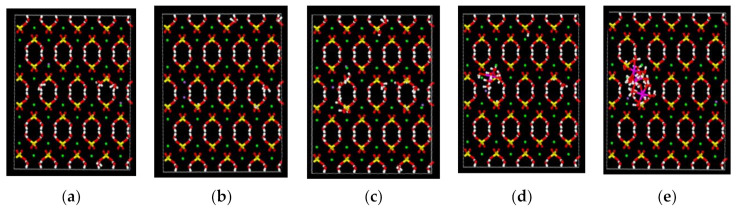
CaSO_4_·2H_2_O surface pores and salt adsorption: (**a**) 2 Å pores, (**b**) 3 Å pores, (**c**) 4 Å pores, (**d**) 5 Å pores, and (**e**) 6 Å pores. (White balls are hydrogen, red balls are oxygen, yellow balls are sulfur, green balls are calcium, purple balls are sodium, and pink balls are aluminum).

In order to explore the effects of surface pores on the mechanical properties of CaSO_4_·2H_2_O, surface pores with different radii were created in the established CaSO_4_·2H_2_O model and the changes in its mechanical properties were studied. Figure 11a–e are models with surface pore diameters of 2 Å, 3 Å, 4 Å, 5 Å, and 6 Å, respectively. The pores are on the left side of the model, and the adsorbed salts can be seen. Table 4 shows the effect of pore size on the mechanical properties of CaSO_4_·2H_2_O crystals. As shown in Table 4, young’s modulus in the X direction of the CaSO_4_·2H_2_O model first decreases and then increases with the increase of pore size, but all models have a larger young’s modulus than the original model. When the pore size is 2 Å, the young’s modulus reaches the peak value of 2.5079 GPa. It can be seen that the pores can enhance the mechanical properties of CaSO_4_·2H_2_O crystals. CaSO_4_·2H_2_O has a layered structure of sulfate and calcium ions with water molecules in between. The porous structure likely has an impact on the layer dynamics during loading. Sarkar et al. studied CaSO_4_·2H_2_O in tensile loading. They discovered that the layer separation decreases after the loading process in the X direction [33]. The formation of pores gives more structural freedom and therefore allows for more molecular rearrangement during loading. This is likely the cause of the increase in young’s modulus. Molecular dynamics simulations show that, within the nano-scale, surface micropores of appropriate sizes can enhance the mechanical properties of CaSO_4_·2H_2_O crystals. In the actual application process, the water–cement ratio of CaSO_4_·2H_2_O is 0.5–0.8, and its theoretical water consumption is only 0.186. After the excess water evaporates, an interconnected pore system is formed, which is the main reason for the decrease in the mechanical properties of CaSO_4_·2H_2_O. CaSO_4_·2H_2_O crystal grains do not contain a large-scale pore system in the nano-scale, so its mechanical properties are higher than the macro-mechanical strength.

## 4. Conclusions

This article explored the influence of sodium carbonate and alum on the performance of phosphogypsum as well as the modification of the nanostructure containing nanopores. Through the changes in the content of sodium carbonate and alum, and the water–cement ratio, the trend and mechanism of phosphogypsum performance were analyzed. The formation and effects of the nanopores were also studied. The conclusions are as follows:

1. Considering the mechanical strength and absolute dry density of phosphogypsum, the best content of sodium carbonate is 0.3% and the best content of alum is 2%. At this time, the initial setting time is 330 s, the final setting time is 390 s, the flexural strength of the phosphogypsum test block is 2.1 MPa, the compressive strength is 7.5 MPa, and the absolute dry density is 990 kg/m^3^.
2. When phosphogypsum is mixed with sodium carbonate and alum, new hydration products are generated, and the CaSO_4_·2H_2_O crystal grains contain a large number of nanopores. Molecular dynamics simulations show that the hydration products are responsible for the surface deformation and the generation of surface nanopores. This is mainly caused by the Van der Waals forces between the Na ions and sulfate groups and by hydrogen bonding of the eroded CaSO_4_·2H_2_O water molecules and NaAl(SO_4_)_2_·6H_2_O. The nanopore structure in the CaSO_4_·2H_2_O crystal yields enhanced mechanical properties by providing structural freedom and molecular rearrangement.

## Figures and Tables

**Table 1:** Contents of sodium carbonate, alum, and water–cement ratios in sample preparation.

No.	Sodium Carbonate Content (%)	Alum Content (%)	Water–Cement Ratio
G_0_	0	0	0.70
G_1_	0	0	0.88
F_1_	0.1	1	0.70
F_2_	0.2	1	0.70
F_3_	0.3	1	0.70
F_4_	0.4	1	0.70
F_5_	0.5	1	0.70
P_1_	0.3	2	0.70
P_2_	0.3	3	0.70
P_3_	0.3	4	0.70
P_4_	0.3	5	0.70
W_1_	0.3	2	0.72
W_2_	0.3	2	0.74
W_3_	0.3	2	0.76

G: the water–cement ratio changed, while the sodium carbonate and alum contents remained at zero; F: sodium carbonate content changed, while the rest remained constant; P: the alum content changed, while the rest remained constant; and W: the water–cement ratio changed, while sodium carbonate and alum contents remained at nonzero constants.

**Table 2:** Effects of different contents of sodium carbonate and alum on properties of phosphogypsum.

No.	Initial Setting Time (s)	Final Setting Time (s)	Flexural Strength (MPa)	Compressive Strength (MPa)	Absolute Dry Density (kg/m^3^)
G_0_	330	598	2.4	8.6	1289
G_1_	530	740	1.7	6.8	993
F_1_	360	540	1.5	7.2	1050
F_2_	390	580	1.9	7.5	1050
F_3_	410	510	2.0	8.1	1030
F_4_	420	560	2.1	7.7	1040
F_5_	490	660	2.3	7.4	1050
P_1_	330	390	2.1	7.5	990
P_2_	210	240	2.2	7.0	1000
P_3_	150	210	1.8	6.0	980
P_4_	120	150	1.7	5.8	1000
W_1_	370	450	2.0	7.2	980
W_2_	410	525	1.8	6.8	972
W_3_	460	590	1.5	6.3	965

**Table 3:** Effects of different content Na_2_SO_4_ and NaAl(SO_4_)_2_·6H_2_O on total internal pore volume.

No.	Na_2_SO_4_ Content (%)	NaAl(SO_4_)_2_·6H_2_O Content (%)	Total Pore Volume (Å^3^)
G0	0	0	97,853.75
F1	0	1	105,481.02
F2	0.1	1	105,747.92
F3	0.2	1	105,676.17
F4	0.3	1	105,965.05
P1	0.3	0	103,818.17
P2	0.3	2	106,379.60
P3	0.3	3	111,369.97

**Table 4:** Effects of pore size on the mechanical properties of CaSO_4_·2H_2_O.

**Pore Size**	0 Å	2 Å	3 Å	4 Å	5 Å	6 Å
**Young’s Modulus (GPa)**	0.73	2.5079	0.8288	1.1416	1.1567	1.7919

## Data Availability

The data presented in this study are available on request from the corresponding author.
